# Anatomical deformation prediction using artificial intelligence for fusion imaging during aortic endovascular procedures

**DOI:** 10.1016/j.jvscit.2026.102297

**Published:** 2026-05-04

**Authors:** Vannier Cindy, Lalys Florent, Hollier-Ben Turkia Jade, Lucas Antoine, Kaladji Adrien

**Affiliations:** a CHU Rennes, Service de Chirurgie Vasculaire, Rennes, France; b Therenva, Rennes, France; c INSERM, U1099, Rennes, France; d Université de Rennes 1, Laboratoire de Traitement du Signal et de l’Image (LTSI), Rennes, France

**Keywords:** Endovascular aneurysm repair, Fusion imaging, Artificial intelligence

## Abstract

Placement of an aortic stent graft induces anatomical deformations, particularly at the iliac level, because of the introduction of rigid devices. Anticipating these changes may improve the accuracy of fusion guidance during endovascular procedures. In this prospective single-center study, a deep learning–based sizing software was used to generate a deformation-adjusted three-dimensional fusion mask, which was compared with a conventional, nondeformed mask using intraoperative angiography as the reference standard. In a cohort of 31 patients, the deformation–adjusted mask significantly improved the fusion accuracy for renal artery ostia positioning, iliac artery alignment, and guidewire localization, without any additional acquisition time. These findings suggest that deformation-aware fusion masks may enhance intraoperative navigation during aortic endovascular repair.

Intraoperative navigation, especially for aortic endografting, has evolved considerably over the past decade with the advent of image fusion, which is now widely used in high-volume aortic centers. This new imaging technique merges preoperative three-dimensional (3D) imaging with two-dimensional (2D) intraoperative fluoroscopy, and, it enables navigation in the vascular tree without the need for regular reinjection of contrast medium.^1^ Image fusion enables important anatomical information, such as the position of the renal and internal iliac arteries, to be superimposed during rigid material placement and stent deployment.^2^ This not only enhances the surgeon's comfort, but also improves technical success by enabling more precise navigation while reducing the dose of X-rays and contrast products required,^3^^,^^4^ especially for complex cases^5^

Placement of an aortic stent graft entails anatomical modifications, particularly at the iliac level, because of the introduction of rigid material.^6^, ^7^, ^8^ These anatomical modifications are the main sources of error in fusion imaging systems, and stenting always requires iterative injection of a contrast medium.

One of the challenges of image fusion is the ability to adapt in real time to anatomical deformations while maintaining the accuracy of landmarks for visceral and internal iliac arteries. Anticipating these deformations could enable more accurate preoperative sizing and smoother navigation.

Some techniques, such as numerical simulation, have been proposed to anticipate these deformations, at the cost of a long calculation time on high-performance machines, making them impractical for use in routine care. Thanks to algorithms incorporating deep learning, it is possible to predict these deformations using the sizing software used in current practice and to generate in near real-time a deformed fusion mask of the images, allowing for precise alignment and improved visualization of anatomical structures. The aim of this study was to evaluate the feasibility and accuracy of the deformation-adjusted 3D fusion mask compared with the undistorted mask with intraoperative angiography.

## Materials and methods

### Study population

The present study was a single-center prospective study conducted at the Rennes Hospital Center from June to December 2024. All patients who met the inclusion criteria were prospectively enrolled: patients considered ineligible for open repair (due to anatomical reasons, depending on their comorbidities or after discussion with the patient about their preferences) and those who had an aneurysm diameter greater than 55 mm for aortic aneurysm and 40 mm for iliac aneurysm. Patients were excluded if their computed tomography (CT) scan could not be adequately analyzed down to the distal iliac arteries, if they already had endovascular material in the iliac arteries, and if they presented with ruptured aneurysms. This study was reviewed by the Research and Innovation Department of Rennes University Hospital and classified as noninterventional research, with all procedures and devices used in accordance with routine clinical practice. Indeed, the procedure followed the standard fusion workflow, with the EndoNaut (Therenva) workstation used in parallel for angiographic recording and deformation analysis.

Written informed consent was obtained from all patients. The study was conducted in accordance with the principles of the Declaration of Helsinki and followed the Strengthening the Reporting of Observational Studies in Epidemiology guidelines for observational studies.

**Fig 1:**
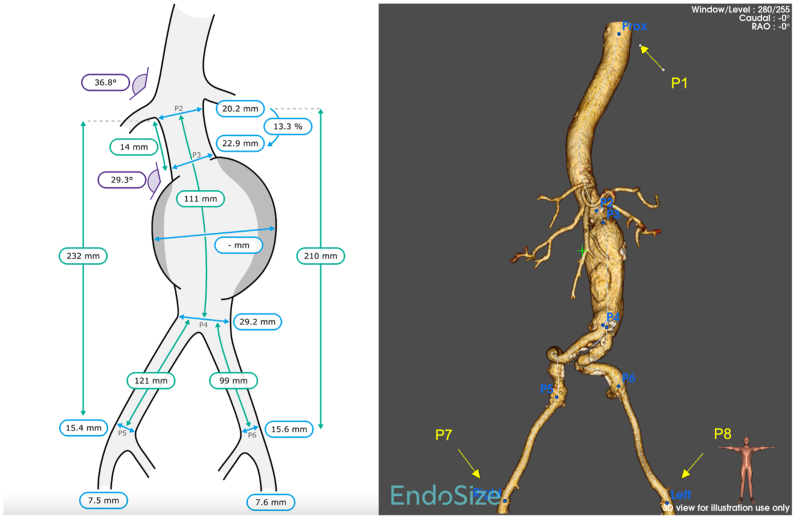
Sizing using EndoSize software with key points.

All preoperative scans were imported into EndoSize software (Therenva) to study patient anatomy in detail. Key points were positioned manually by the surgeon to define the centerline: at the level of the thoracic aorta for P1, distal to the external iliac arteries for P7 on the right and P8 on the left. P2 was then added below the lowest kidney, P3 on the distal part of the aortic neck, P4 at the aortoiliac bifurcation, P5 and P6 at the right and left bifurcations, respectively, in cases of abdominal aortic aneurysms (Fig 1). For fenestrated stents, key points were added opposite to the celiac trunk, superior mesenteric artery, and renal arteries. Within the aorta, thrombus and lumen were semiautomatically segmented from P2 to P4 using a module integrated into EndoSize, and calcifications were segmented automatically. For each iliac axis (ie, P4-P7 on the right and P4-P8 on the left), total length, tortuosity index, thrombus, and calcification volume were extracted.

### Generation of fusion mask

Real-time estimation of arterial deformations was obtained on the basis of the recent work of Mozahem et al^9^ who proposed an updated parametrization of a biomechanical finite element (FE) model to simulate the vascular structure deformation during endovascular aneurysm repair (EVAR). Although this novel FE model demonstrates efficacy in predicting renal ostia displacement and iliac artery deformations during the procedure, the time required to compute predictive models remains high (>3 hours) and is not conducive to routine clinical practice. Therenva proposed a deep-learning model to accelerate this computation time (patent number EP2025069369). A database comprising 250 CT scans of patients with abdominal aortic aneurysm was obtained, and FE simulations were initiated for each patient to generate pairs of nondeformed and deformed anatomies, along with the corresponding 3D deformation fields. From these pairs, offline, 200 were used to train a deep learning model, and 50 were retained for the purpose of validation. The model was constructed using the raw CT and segmented arteries as inputs, and the deformation fields were generated as outputs and used for creating the deformed anatomy. Using the most suitable model derived from the validation set, EndoSize is capable of rapidly acquiring the deformed anatomy of each new patient, facilitating its incorporation into fusion imaging alongside the nondeformed anatomy.

### Fusion imaging

**Fig 2:**
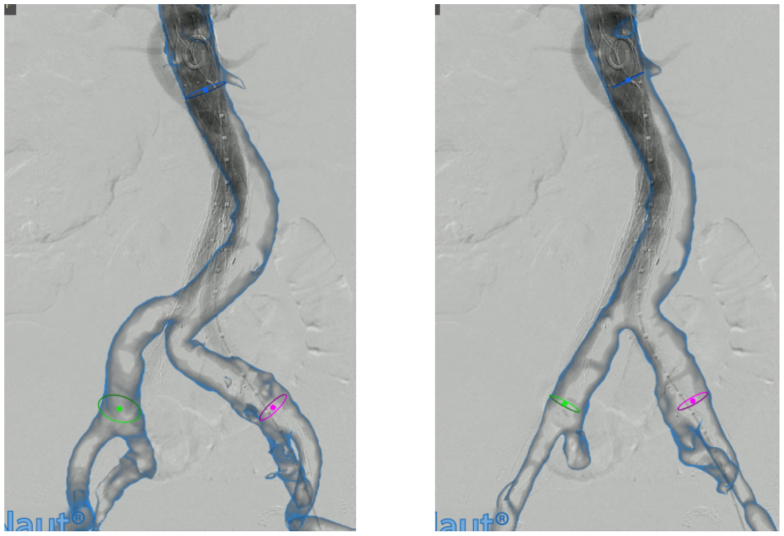
Fusion overlay at the iliac level using an undeformed fusion mask (*left*) and a deformed fusion mask (*right*).

Image fusion was performed using the EndoNaut station, enabling 3D/2D synchronization between preoperative CT and intraoperative fluoroscopy. Device sizing was performed using EndoSize, and the generated fusion masks were subsequently exported to the EndoNaut fusion station along with the preoperative scanner (Fig 2). The 3D fusion mask was obtained using bony and arterial landmarks, with the circles representing the ostia of the renal and internal iliac arteries. Angiography was performed using a pigtail catheter on the renal arteries and each hypogastric artery, with the stiff device in place (Lunderquist guidewire; Cook Medical) to check the accuracy of the fusion mask. All intraoperative images were automatically recorded at native resolution to accurately analyze the deformation of angiographic vascular structures for comparison with undeformed and deformed fusion masks.

### Data analysis and objectives

**Fig 3:**
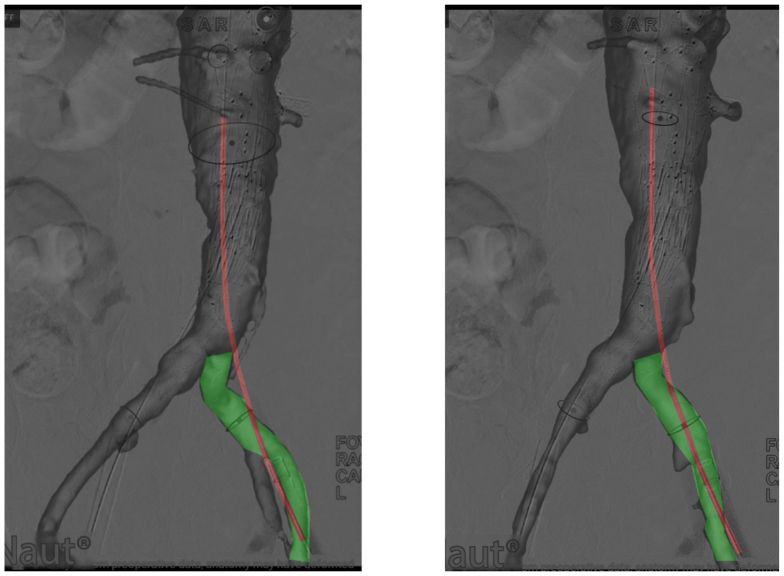
Manual segmentation of the external iliac artery and guidewire.

The primary objective was to assess the accuracy of the deformed fusion mask by comparing it with the nondeformed fusion mask currently in practice. To quantify the accuracy of the deformed versus nondeformed fusion mask, intraoperative angiography was used as a reference. The comparison of coordinate and segmentation calculations between the deformed and undeformed masks and the angiography was carried out using MATLAB software (MathWorks). Four criteria were used to compare certain points of the deformed (Def) and nondeformed (Nodef) 3D mask with angiography (Art): position of renal and hypogastric ostia, percentage of guidewire that passed through the iliac (%gui), and Hausdorff distance (Hd) (mean of minimum distances) between iliac arteries. The coordinates of the key points were calculated on the fusion masks and then converted into millimeters using an average resolution obtained from the average distance between the patient and the image amplifier placed on the table. To determine the percentage of guidewire in the artery, a manual segmentation of each iliac and corresponding guidewire was then carried out (Fig 3); the greater this proportion, the closer the induced deformation is to that observed on angiography. The secondary objective was to analyze, on the preoperative CT scan, the anatomical factors negatively affecting the accuracy of the fusion mask.

### Statistical analysis

Descriptive statistics, including means, standard deviations, ranges, and proportions, were calculated using MedCalc Statistical Software (Ostend). Results were expressed as mean if the normality test was accepted on the Shapiro-Wilk test; otherwise, they were expressed as median with confidence interval. Univariate analysis was also conducted to identify potential anatomical factors associated with fusion error. Standard Pearson's rho correlation with associated *P* value was used.

All statistical analyses were performed using R software (R Foundation for Statistical Computing). A *P* value <.05 was considered statistically significant.

## Results

### Patient characteristics

Patients undergoing EVAR (*n* = 10, 32.25%), EVAR and iliac branched endografts (*n* = 6, 19.35%), fenestrated EVAR (*n* = 12, 38.7%), fenestrated EVAR with EVAR and iliac branched endograft (*n* = 2, 6.45%), and branched EVAR (*n* = 1, 3.25%) for aneurysm were prospectively included. A total of 31 patients (77% men; mean age 72.6 years with range 64-89; mean body mass index 25.5) were enrolled. Fusion imaging was possible in all included patients. Patients mainly received endografts manufactured by Cook Medical (*n* = 22, 71%), followed by Medtronic (*n* = 6, 19%), and Gore (*n* = 3, 10%). Patient characteristics are summarized in Table I.

**Table I:** Population characteristics (*n* = 31)

Characteristic	Value
Clinical characteristics	
Age, years	72.6 ± 6.1
Male sex	24 (77.4)
Body mass index, kg/m^2^	25.5 [24.1-27.9]
Comorbidities (history of)	
Smoker (current smoker)	12 (38.7)
History of smoking	15 (48.4)
Hypercholesterolemia	21 (67.7)
Diabetes	4 (12.9)
Peripheral artery vascular disease	8 (25.8)
Hypertension	19 (61.3)
Coronaropathy	6 (19.6)
Stroke	1 (3.2)
Chronic kidney disease	7 (22.6)
Current medications	
Statins	22 (71.0)
Antihypertension treatment	21 (67.7)
Antiplatelet	21 (67.7)
Antidiabetics treatment	6 (19.3)
Intended endovascular device	
Cook Medical	22 (71)
Medtronic	6 (19)
Gore	3 (10)
Types of procedures	
EVAR	10 (32.2)
FEVAR	12 (38.7)
EVAR and iliac branched	6 (19.3)
FEVAR and iliac branched	2 (6.5)
Other	1 (3.3)

*EVAR*, Endovascular aneurysm repair; *FEVAR*, fenestrated endovascular aneurysm repair.

Variables are presented as n (%), mean ± standard deviation, or median [95% confidence interval].

### Procedural details

All endografts were deployed over rigid Lunderquist guidewires. The mean fluoroscopy time was 48 minutes (range 8-121 minutes), the amount of contrast medium used was 66.6 (range 27-116 mL), median dose-surface product was 34.4 Gy·cm^2^ (range 7.78-153.3) (Table II).

**Table II:** Intraoperative data

Variable	Value
Contrast, mL	66.6 ± 24.9
EVAR	51.2 ± 22.4
FEVAR	79.2 ± 15
Branched iliac + EVAR or FEVAR	59.5 ± 27.1
Other	110
Fluoro time, min	48 ± 25.2
EVAR	22.4 ± 10.7
FEVAR	62.8 ± 10.3
Branched iliac + EVAR or FEVAR	45.4 ± 18.4
Other	121
DAP, Gy·cm^2^	34.4 [27.4-49.1]
EVAR	36 [14.03-57.77]
FEVAR	34.84 [29.76-50.00]
Branched iliac + EVAR or FEVAR	31.15 [20.17-79.20]
Other	64.06

*DAP*, Dose-area product; *EVAR*, endovascular aneurysm repair; FEVAR, fenestrated endovascular aneurysm repair; *IQR*, interquartile range; *SD*, standard deviation.

Variables are presented as mean ± SD or median [95% confidence interval].

### Primary objective

The time taken to generate the Def in EndoSize was <45 seconds per patient. A significant difference was found with a smaller distance to key markers on angiography when the distorted fusion mask was used for renal artery ostia position, Hausdorff distance, and percentage of guidewire in the artery (Table III).

**Table III:** Ostia and guide metric comparison

Parameter	Undeformed mask	Deformed mask	*P* value
Renal artery ostia distance, mm			
Total distance	5.7 ± 4.3	3.4 ± 2.7	<.001
Left renal	5.7 ± 4.1	3.4 ± 2.6	<.001
Right renal	5.7 ± 4.5	3.3 ± 2.9	<.001
Internal iliac ostia distance, mm			
Total distance	8.7 ± 7.8	8.6 ± 6.7	.90
Left iliac	8.3 ± 7.7	8.0 ± 6.3	.84
Right iliac	9.1 ± 8.0	9.2 ± 7.2	.93
Hausdorff iliac distance, mm			
Total distance	2.7 ± 0.7	2.2 ± 0.5	<.001
Left iliac	2.7 ± 0.7	2.3 ± 0.5	<.002
Right iliac	2.6 ± 0.7	2.2 ± 0.5	<.007
Percentage of guidewire within the iliac artery			
Guide	46.4 ± 19.5	57.9 ± 17.1	<.001
Left guide	44.1 ± 19.6	54.7 ± 14.9	.01
Right guide	49.0 ± 19.5	61.3 ± 18.8	.006

The distance of the renal artery ostia between intraoperative arteriography (Art) and undeformed mask (Nodef) was 5.7 ± 4.3 mm and 3.4 ± 2.7 mm between Art and Def (*P* < .001). The distance of the hypogastric ostia between Art and Nodef was 8.7 ± 7.8 and 8.6 ± 6.7 mm between Art and Def (*P* = .9). The %gui was 46.4 ± 19.5% on the Nodef mask and 57.9 ± 17.1% on the Def mask (*P* < .001). The Hausdorff distance was 2.7 ± 0.7 mm on the Nodef mask and 2.2 ± 0.5 mm on the Def mask (*P* < .001).

### Secondary objective

The amount of thrombus at the aortic level shows a correlation with fusion error at both renal and iliac levels (*P* = .01 and *P* = .002, respectively), whereas the amount of iliac thrombus shows a correlation only at the level of fusion error at the iliac floor (*P* = .002 on the right iliac and *P* = .005 on the left iliac). Both right and left iliac tortuosities correlate with fusion errors at the iliac and aortic levels. The tortuosity ratio between P2 (subrenal neck) and points P5 and P6 (distal neck) correlates with fusion errors at the iliac level. Infrarenal angulation and aneurysm diameter correlate with iliac error (*P* = .007 and *P* = .02, respectively). In contrast, calcification volume showed no correlation with fusion errors (Table IV).

**Table IV:** Discrepancy between the distorted fusion mask and angiography

Parameter	Correlation with errors on renals^a^	Correlation with errors on iliacs^a^
Aortic parameters		
Aneurysm diameter	0.1901	0.5092 (0.007)
Aortic neck length	0.1517	0.2389
Suprarenal neck angle	0.5012	0.1583
Infrarenal neck angle	0.2547	0.4385 (0.02)
Aortic lumen	0.1175	0.1027
Aortic thrombus	0.4813 (0.01)	0.5576 (0.002)
Aortic calcification volume	0.0855	0.0655
Aortic calcification	0.1804	0.1031
Tortuosity index		
P2-P3	0.2233	0.0515
P2-P4	0.0556	0.2662
P4-P5	0.4459 (0.02)	0.0155
P4-P6	0.3667	0.4257 (0.02)
P2-P5	0.4895 (0.01)	0.3600 (0.06)
P2-P6	0.1907	0.5391 (0.003)
Iliac parameters – right iliac		
Tortuosity	0.5052 (0.008)	0.4233 (0.02)
Thrombus volume	0.3498	0.5737 (0.002)
Calcification, mm^3^	0.0136	0.0992
Calcification, %	−0.2295	−0.1690
Iliac parameters – left iliac		
Tortuosity	0.4154 (0.04)	0.6816 (0.0001)
Thrombus volume, mL	0.1786	0.3827 (0.05)
Calcification, mm^3^	0.1225	0.2306
Calcification, %	0.1032	0.1447

a Standard Pearson's rho correlation with associated *P* value in parentheses when significant.

## Discussion

Fusion imaging can be integrated into daily clinical routine; however, the introduction of stiff device during endovascular procedures sometimes can lead to significant anatomical deformations, as shown by Schulz et al,^7^ which is one of its main limitations. Although image fusion is intended to allow rapid and accurate repositioning during the procedure, our study suggests that fusion with the deformed mask is both feasible and more accurate than with a mask not adapted to anatomical deformations.

The position of the renal arteries is crucial to the deployment of the stent in order to avoid covering them. In our series, the insertion of rigid material results in a mean error of displacement of the renal artery ostium of 5.1 ± 4.3 mm for the undeformed mask and 3.4 ± 2.1 mm for the deformed fusion mask (*P* < .001). In comparison, the lateral and craniocaudal deviation of the lowest renal artery ostium was a median of 6 mm in the study of Schulz et al^7^ on 101 EVARs; other studies with smaller numbers found variable results, with median errors ranging from 1 to 2.2 mm.^10^, ^11^, ^12^, ^13^, ^14^ The median deviation was 4.1 ± 2.4 mm in a study previously conducted from a cohort of the Rennes vascular surgery department, on undistorted fusion masks.^12^ In addition, several studies^12^^,^^15^^,^^16^ have also shown consistent deformation of the aorta in the visceral aortic segment, with an average displacement of 7.8 mm in a study of 77 vessels.^14^ However, none of these studies address the estimated displacement at the level of the internal iliac arteries. In our study, a greater distance from arteriography was observed at the level of the internal iliac artery ostia compared with the renal artery ostia, reflecting the difficulty in obtaining an accurate fusion mask at the level of the iliacs. It can be hypothesized that these results are related to the difficulty in properly clearing the internal iliac ostia, often requiring greater oblique and caudal angulations than at the renal level.

We also investigated which preoperative anatomical parameters were the source of a significant discrepancy between the distorted fusion mask and the angiography. In our study, different anatomical factors appear to influence fusion errors, including the amount of aortic thrombus and vessel tortuosity. In a retrospective article analyzing 41 patients,^15^ iliac lengths were significantly correlated with the total deformation level, highlighting the fact that aortic morphology influences its deformation. In the study by Jansen et al,^14^ involving 14 patients and 77 target vessels, the maximum tortuosity angle appeared to be related to the extent of target vessel displacement. In our series, infrarenal angulation also appears to be a parameter that contributes to errors, and this result corresponds to the study by Lalys et al,^6^ who showed in a multivariate analysis that only the angulation between the neck and the aneurysm sac had an impact on the accuracy of fusion imaging; the other parameters studied showed no correlation. The volume of calcifications does not seem to correlate with fusion errors at either aortic or iliac level; these results are comparable with those found in the literature.^6^^,^^7^

Our results demonstrate a reduced distance to key markers, thanks to the use of the deformed fusion mask. In current practice, the use of iodinated contrast agent remains necessary to verify the good precision of the mask before deploying the stent. The future objective is to achieve a procedure without injection of contrast medium, thanks to a lack of fusion perfectly adapted to the deformed anatomy of each patient. The end points evaluated in this study should be interpreted as surrogate markers reflecting technical accuracy rather than direct clinical benefit. The improvement in deformation prediction and angiographic concordance suggests the potential of artificial intelligence (AI)–based CT fusion. To demonstrate a reduction in contrast agent use, radiation exposure, procedure time, or an improvement in deployment accuracy, prospectively designed studies specifically designed for this purpose would be required. Our results therefore represent a first step toward this goal.

## Limitations

This study has several biases: first, its monocentric nature. Second, manual positioning of key points and semiautomatic segmentation of thrombus volume were performed by a single operator, which could lead to bias in the results.

Owing to technical limitations, we did not take into account other possible etiologies of fusion error, such as patient movement. In addition, the anticipation of deformations does not take into account the posterior anterograde displacement of the aorta and the renal and hypogastric arteries.

In the future, the study of more complex cases and larger patient samples will enable image fusion to be refined, particularly in the case of thrombi or iliac tortuosities.

## Conclusions

Real-time adaptation to anatomical deformations during the introduction of rigid material remains a major challenge for endovascular procedures. This study shows that with an algorithm integrated into a sizing software used in daily practice, it is possible to easily generate an image fusion mask that is more accurate than the usual undistorted fusion mask. A variety of more anatomically complex cases will improve the performance of this anticipation.

## Funding

This study did not receive any specific funding from public, commercial, or not-for-profit funding agencies.

## Declaration of generative AI and AI-assisted technologies in the writing process

During the preparation of this work, the authors used DeepL Translate (Deepl SE: https://www.deepl.com/translator) for translation of text passages. After using this tool, the authors reviewed and edited the content as needed and take full responsibility for the content of the publication.

## Disclosures

Florent Lalys is employed by Therenva. Adrien Kaladji reports proctoring fees from Cook Medical and consulting fees from Biotronik and Medtronic. The other authors declare no conflicts of interest related to this work.
